# 165 Higher level of learning – evaluation of the effects of introducing standing desks for part of school children’s lessons

**DOI:** 10.1093/eurpub/ckae114.171

**Published:** 2024-09-26

**Authors:** Piotr Matłosz, Jarosław Herbert, Alejandro Martinez-Rodriguez, Justyna Wyszyńska, Liliya Morska

**Affiliations:** Institute of Physical Culture Sciences, Medical College, University of Rzeszów, Rzeszów, Poland; Institute of Physical Culture Sciences, Medical College, University of Rzeszów, Rzeszów, Poland; Analytical Chemistry, Nutrition and Food Science Department, University of Alicante, Alicante, Spain; Institute of Health Sciences, Medical College, University of Rzeszów, Rzeszów, Poland; Institute of Pedagogy, College of Social Sciences, University of Rzeszów, Rzeszów, Poland

## Abstract

**Purpose:**

This is the first study of Polish children in which both the psychological and physiological effects of the use of standing desks in the classroom have been assessed. The study evaluated cognitive skills, posture, body composition, and sleep quality before and after introducing standing desks in school classrooms for four weeks.

**Methods:**

The study involved 54 male students, aged 11 to 13, from a primary school.

The study procedure has three assessment points: the first at the beginning of the school semester in February, the second after four weeks of standard classes, and the third after four weeks of using standing desks for half of each lesson.

The cognitive skills were assessed using the D2 and Stroop tests. Body composition was evaluated using the Tanita 420BC analyzer. Foot static loads were measured with the FreeMed Base tensometric platform. Body posture was evaluated using the Infrared-based 3D body scan Kineod System. Physical activity and sleep were assessed with Actigraphy monitors, specifically the ActiGraph GT3X-BT Monitor.

**Results:**

Significant changes in anthropometric variables and body composition were observed at all three measurement points. No significant differences were observed in body posture, static foot loads, or sleep parameters. After the introduction of standing desks, significantly less light physical activity and more vigorous physical activity were observed. Children had significantly better results on cognitive tests when working at standing desks compared to standard sitting desks.

**Conclusions:**

Changes in physiological parameters observed may be due to normal physiological development in children during ontogenesis. However, improved physical activity and cognitive skills suggest that standing desks could be successfully introduced in the school environment to enhance learning.

